# Colon fibroblasts from Pirc rats (F344/NTac‐*Apc* ^am1137^) exhibit a proliferative and inflammatory phenotype that could support early stages of colon carcinogenesis

**DOI:** 10.1002/ijc.33796

**Published:** 2021-09-18

**Authors:** Katia Tortora, Francesca Margheri, Cristina Luceri, Alessandra Mocali, Sara Ristori, Lucia Magnelli, Giovanna Caderni, Lisa Giovannelli

**Affiliations:** ^1^ NEUROFARBA Department, Pharmacology and Toxicology Section University of Florence Florence Italy; ^2^ Department of Experimental and Clinical Biomedical Sciences Mario Serio University of Florence Florence Italy

**Keywords:** *Apc*, CAFs, colon cancer, cytoplasmic chromatin foci, DNA damage response

## Abstract

The role of fibroblast *APC* mutation in carcinogenesis is not clear. *Apc* ^+/−^ colon fibroblasts have been previously characterized: however, little is known about their behavior at very early‐stage of colon carcinogenesis. We cultured colon mucosa fibroblasts (PCF, *Apc* ^+/−^) of Pirc rats (F344/NTac‐*Apc* ^am1137^) at an early stage of tumorigenesis, in absence of preneoplastic lesions, and of age‐matched wt (WCF): DNA damage levels, inflammatory phenotype and the expression of known markers of CAFs were analyzed. The latter were also assessed by microarray analysis on colon normal mucosa of Pirc and wt animals. PCF exhibited higher proliferative rates (*P* < .001) and delayed replicative senescence onset (*P* < .05) compared to WCF, along with a lower level of oxidative DNA damage (*P* < .05). Furthermore, a constitutively higher expression of COX‐2 and sensitivity to inflammatory stimuli was found in PCF compared to WCF (*P* < .05), accompanied by higher invasive capability (*P* < .05) and presence of cytoplasmic chromatin foci (cytoplasmic chromatin foci, *P* < .05). However, they neither expressed CAFs markers (α‐SMA, IL‐6) nor responded to CAFs activating stimuli (TGF‐β). Accordingly, CAFs markers and activating stimuli resulted down‐regulated in Pirc normal mucosa compared to wt, whereas DNA damage response and tolerance pathways were overexpressed. These data show for the first time that a proliferative and inflammatory phenotype characterizes *Apc* ^+/−^ colon fibroblasts since very early stages of colon tumorigenesis, and indicate a role of *Apc* mutation in driving fibroblast phenotypic alterations that could support the establishment of a protumorigenic environment. Early pharmacological targeting of these dysfunctions might impact on tumor prevention in FAP patients.

## Abstract 2

What's new?

Heterozygous mutations in *APC* represent the earliest event in sporadic colorectal carcinogenesis onset and cause familial adenomatous polyposis syndrome. However, the role of *APC*‐mutated fibroblasts remains unclear. Here, *Apc* ^+/‐^ fibroblasts isolated from apparently‐normal colon tissue of Pirc rats showed proliferative, inflammatory features and resistance to oxidative DNA damage, although they did not show cancer‐associated fibroblast features. These data suggest that, at the very early stages of colon tumourigenesis, *Apc*‐mutated colon fibroblasts favour the establishment of a pro‐tumourigenic environment for pre‐neoplastic lesion development. Early pharmacological targeting of these dysfunctions might be valuable for tumour prevention in familial adenomatous polyposis patients.

**Abbreviations**
*Apc*
: adenomatous polyposis coli
CAFs: cancer‐associated fibroblasts
FAP: familial adenomatous polyposis
HR: homologous repair
IF: immunocytofluorescence
LPS: lipopolysaccharide
MDF: mucin depleted foci
NFkB: nuclear factor kappa‐light‐chain‐enhancer of activated B cells
PCF: Pirc colon fibroblasts
Pirc: polyposis in the rat colon
TGF‐β: transforming growth factor beta
TNF‐α: tumor necrosis factor alpha
WCF: wild type colon fibroblasts
α‐SMA: alpha‐smooth muscle actin

## 1 INTRODUCTION

*APC* is a tumor suppressor gene encoding for a protein involved in controlling the WNT signal transduction pathway, cell adhesion, migration, apoptosis, inhibition of the DNA base excision repair system (BER), transcriptional regulation and chromosomal segregation at mitosis.^1^, ^2^ Heterozygous mutations in *APC* represent the earliest event in sporadic colorectal carcinogenesis (CRC) onset and cause familial adenomatous polyposis syndrome (FAP), a heritable disease that increases the risk of colon cancer development.^3^ Although prophylactic colectomy is performed (mean age of 20 in Europe) to avoid this event, the risk of cancer onset in the rectum remains, together with comorbidities and development of desmoid tumors (localized intraabdominal or on the back) whose removal is often accompanied by complications.^3^, ^4^ Fibroblasts from FAP individuals' skin and desmoid tumors have been characterized before, showing growth abnormalities, high invasiveness and no susceptibility to DNA damaging agents.^5^, ^6^, ^7^

Fibroblasts are quiescent cells that become activated in response to wounds in order to maintain tissue homeostasis thus showing higher proliferation, increased secretion of ECM proteins to remodel tissue microenvironment, and augmented release of signaling factors.^8^ In turn, tumor cells release signaling molecules such as TGF‐β, interleukins and PDGFs to activate resident fibroblasts into cancer associated fibroblasts (CAFs).^9^

Beside the features listed above, CAFs are characterized by an enriched secretome, including growth factors, cytokines, chemokines and interleukins (ie, IL‐6) accounting for the negative correlation between CAFs percentage and prognosis for several cancers.^10^, ^11^ They are usually identified using VIMENTIN, S100A4 (fibroblast specific protein), α‐SMA (α‐smooth muscle actin) and FAP (fibroblast activating protein) as markers.^12^ Notably, CAFs are not just endured by cancer cells but they can specifically be affected by genetic and genomic alterations and could have a different behavior depending on the nature of the mutation, sporadic or heritable.^13^, ^14^

A proteomic analysis was performed on colon fibroblasts isolated from apparent normal mucosa of FAP patients (*APC* ^+/−^) or from sporadic colon cancer: interestingly, changes observed in those fibroblasts were shared by epithelial cells isolated from colonic crypts in the same subgroups of patients, and an enhanced oxidative stress response was found in both fibroblasts and epithelial cells mutated in *APC*.^15^, ^16^ Another study exploited differential gene expression of fibroblasts isolated from the preneoplastic lesions aberrant crypt foci (ACF) mutated in *KRAS*, *BRAF* or *APC*: genes encoding secreted proteins with fibroblast activating functions were moderately upregulated in all three types of mutated ACF epithelial cells compared to respective normal mucosa.^17^ Interestingly, senescence was particularly evident in *BRAF* and *KRAS* mutated ACF but not in *APC* mutated ones. All in all, the role of fibroblast *APC* mutation in both colon carcinogenesis and desmoid tumor onset still needs to be elucidated.

The use of Pirc rat (F344/NTac‐*Apc* ^am1137^), a genetic model of *Apc*‐driven CRC developing preneoplastic lesions such as mucine depleted foci (MDF) and adenomas in the colon, allows the study of tumorigenesis during its evolution from the absence of lesions, to the development of MDFs and then macroscopic adenomas.^18^, ^19^ Besides, Pirc colon mucosa shows different features compared to age matched wt rats even in absence of preneoplastic lesions.^18^, ^20^

The aim of our study was to characterize *Apc* mutated colon fibroblasts isolated from Pirc colon mucosa at an early stage of CRC, to gain information on the interplay between epithelial cells and fibroblasts in absence of preneoplastic lesions. Thus, we used 1‐month‐old Pirc rats, not yet bearing MDFs, and age matched wt animals to establish colon fibroblast primary cultures (PCF and WCF, respectively).

## 2 MATERIALS AND METHODS

### 2.1 Pirc and wt colon fibroblast primary cultures establishment

Colon fibroblasts were isolated from Pirc (PCF) and wt (WCF) rats of 1 month of age (n = 3/group). All procedures were in accordance with EU Directive 2010/63/EU for animal experiments. The entire colons were washed and samples from medial normal mucosa of approximately 2 cm^2^ each were collected and transferred in sterile tubes containing 40 mM DTT in HBSS. Samples were washed with HBSS/PSG (penicillin 100 U/mL, streptomycin 0.1 mg/mL and gentamycin 50 μg/mL) under sterile atmosphere, quickly cut into small pieces and then transferred in sterile tubes for enzymatic digestion (30 min in a water bath at 37°C under shaking) with 1 mg/mL collagenase II (Sigma Aldrich, Milan, Italy) dissolved in advanced DMEM plus 1% antibiotic‐antimycotic solution (both purchased from GIBCO), and 3 mM CaCl_2_. Digested samples were recovered, transferred in new tubes containing advanced DMEM supplemented with 20% FBS, 1% antibiotic‐antimycotic solution, 1% l‐glutamine and then centrifuged three times at 1200 rcf for 5 min at RT, discarding the supernatant each time. Finally, fragments were resuspended in the same media, seeded in a 6‐MW plate and incubated in a humidified atmosphere in presence of 5% CO_2_. Plates were daily checked for contamination until groups of cells were visible in proximity to tissue fragments. These were completely removed as soon as 50% of the surface was covered by cells and FBS supplementation was decreased from 20% to 10% at the first subculture.

### 2.2 Cell growth

Cell growth was assessed performing MTS viability test accordingly with the manufacturer instructions (Promega, Gessate, Italy). Cells were seeded in 96‐MW (3000 cells/well) and viability was measured at 24, 48 and 72 hr after seeding (T_0_, T_24_ and T_48_, respectively). Data were plotted as a function of % viability from T_0_ considered as 100%.

### 2.3 Immunocytofluorescence and immunohistochemistry

Immunocytofluorescence (IF) and immunohistochemistry (IH) were performed as previously described (see Appendix S1 for details).^21^, ^22^

### 2.4 Senescence associated β‐galactosidase activity

As for IF assay, cells were seeded on coverslips in 24‐MW plates and fixed with a 3% formaldehyde solution. Then, they were incubated in the dark at 37°C for 5 hr with the staining solution prepared as reported in literature by Dimri et al.^23^ At least six microscopic fields/slide were captured (×20 magnification) with an optical microscope. Differences in staining intensity rather than positive cells for β‐galactosidase were noticed in preliminary experiments (data not shown), thus we quantified staining intensity instead of counting positive cells. Staining intensity was expressed as the mean gray value calculated with the use of ImageJ software.

### 2.5 Ex‐vivo of Pirc adenomas in conditioned medium from PCF and WCF

Ex‐vivo system of adenomas samples from 11 months old Pirc rats (n = 2) were established as previously described (see Appendix S1 for details).^22^

### 2.6 Western blotting assay on PCF and WCF after proinflammatory stimulation

PCF and WCF were seeded in 6‐MW plate (50 000 cells/well) and treated for 24 hr with advanced DMEM supplemented with 1% FBS, 1% l‐glutamine, 1% antibiotic‐antimycotic and: (a) 10 μg/mL LPS; (b) 20 ng/mL TNF‐α or (c) 10 ng/mL TGF‐β. At the end of treatments, proteins from monolayers were extracted, run and bands acquired and quantified as previously described (see Appendix S1 for details).^22^, ^24^

### 2.7 Il‐6 ELISA assay

Media from the treatments illustrated in the previous paragraph were collected, centrifuged for 5 min at 1200 rcf and supernatants aliquoted and stored at −20°C until use. IL‐6 was assessed by ELISA according to the kit manufacturer instructions (Rat IL‐6 Mini ABTS ELISA Development kit, Peprotech).

### 2.8 Spontaneous invasion assay

This test was performed in Boyden chambers, with wells separated by 8 μm‐pore size polycarbonate filters coated with Matrigel (50 μg/filter). Subconfluent PCF and WCF cultures of 2 × 10^4^ cells were suspended in 200 μL of fresh Advanced DMEM plus 2% FBS and placed in the upper wells of Boyden chambers. Fresh DMEM plus 2% FBS was also placed in the lower well. After 24 h at 37°C in 5% CO_2_, filters were recovered, fixed in methanol and stained in order to count cells under a light microscope.

### 2.9 Comet assay

The comet assay was performed, as previously described,^25^ in order to assess basal levels of DNA breaks and DNA base oxidation in PCF and WCF (see Appendix S1 for details).

### 2.10 Microarray on normal mucosa from 1‐month‐old Pirc and wt rats

Samples of scraped normal colon mucosa of 1‐month‐old male Pirc and wt rats were harvested at sacrifice, and stored individually at −80°C in RNAlater (Qiagen, Milan, Italy). Total RNA was isolated with the RNeasy Mini kit the NanoPhotometer spectrophotometer (IMPLEN).

The expression profiles of eight Pirc colon mucosa (cy5‐labeled) were compared to that of a pool of RNAs from samples of WT rat colon mucosa (cy3‐labeled). To produce Cy3 and Cy5‐labeled cRNA, 100 ng of each RNA sample were labeled using the Agilent Quick Amp Labeling Kit (Agilent Technologies). Yields of cRNA and the dye‐incorporation rate were measured with the NanoPhotometer. RNA Spike‐in were also used to monitor and calibrate the linearity, sensitivity and accuracy of the microarray workflow. The labeled samples were hybridized to Agilent SurePrint G3 rat GE 8x60K microarrays, in Agilent microarray chambers, at 65°C for 18 h. Fluorescent signal intensities were detected by using the Agilent Scan Control 7.0 Software on an Agilent DNA Microarray Scanner, at a resolution of 2 μm. Data were acquired using Agilent Feature Extraction 9.5.3.1 software and values for control spots and spots that did not meet the quality criteria were flagged.

Differences in gene expression between Pirc and WT colon mucosa were analyzed using a t‐moderated test with the Benjamini‐Hochberg correction of the FDR (false discovery rate) for multiple tests. Genes with a FDR‐adjusted *P*‐value of less than .05 were considered differentially expressed.

### 2.11 Statistics

Data from MTS, ß‐galactosidase, cytoplasmic chromatin foci, COMET assay, western blotting for COX‐2 and α‐SMA, were analyzed by two‐way ANOVA (GraphPad Prism 5.0) followed by Bonferroni's posttest, in order to consider the effect of both the specific variable associated to the assay (passages in culture, incubation with FPG enzyme and treatments, respectively), and the genotype of the cells (Pirc vs wt). Overlapping coefficient and integrated density on NFkB, β‐catenin and γ‐H2Ax images, respectively, were calculated with ImageJ software, followed by analysis with the Student *t*‐test for unpaired measures. This test was also used for analyzing data from ELISA, α‐SMA expression and invasion assay. Data from ex‐vivo experiments with PCF and WCF conditioned media were analyzed by one‐way ANOVA followed by Bonferroni's posttest.

## 3 RESULTS

### 3.1 Proliferative activity and replicative senescence in *Apc* mutated colon fibroblasts

**FIGURE 1:**
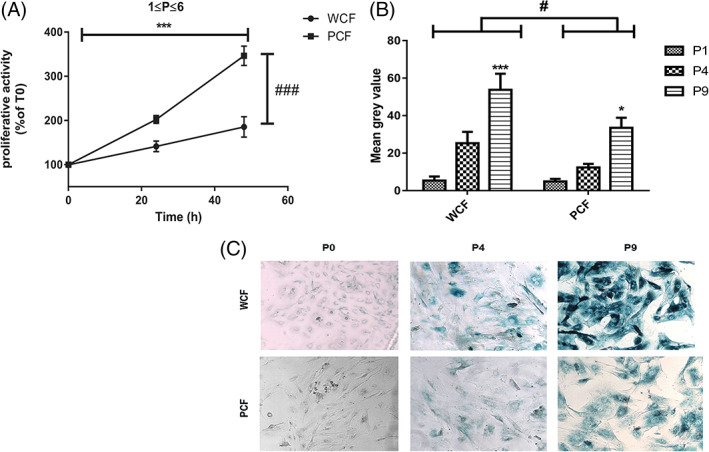
Proliferative activity and replicative senescence onset of PCF and WCF. (A) Proliferative activity was evaluated by MTS assay performed 24 h (T_24_) and 48 h (T_48_) in WCF and PCF between passages (P) 1 to 6 (mean ± SE, n = 5 independent experiments on three couples of matched WCF and PCF). *** and ^###^ *P* < .001 considering both time and genotype by two‐way ANOVA analysis. (B) β‐Galactosidase staining intensity was expressed as mean gray value measured by ImageJ software in at least five microscopic fields/passage for both WCF and PCF (mean ± SE, n = 6 for both groups at P1, n = 12 for both groups at P4 and n = 20 for both groups at P9). ^#^ *P* < .05 significantly different from WCF; *, ** *P* < .05 and .01, respectively significantly different from P1. (C) Representative images for β‐galactosidase at P0, P4 and P9 in WCF and PCF (×40 magnification) [Color figure can be viewed at wileyonlinelibrary.com]

Proliferative activity of PCF and WCF, measured by MTS cell viability test, was assessed between 0 and 6 passages in culture (P) and between P7‐9: the reason of this choice was that a reduction in mitosis counts was observed in P7 cells, more pronounced in WCF than PCF, leading us to consider them separately (data not shown). As shown in Figure 1A, the number of viable PCF cells almost duplicated at T_24_ and further increased at T_48_ indicating a significant higher proliferative activity compared to WCF at both time points (*P* < .001, n = 5). However, both WCF and PCF proliferation slowed down at increased passages in culture (data not shown), suggesting the possible onset of *in vitro* replicative senescence. To verify this hypothesis, we assessed the expression of the senescence marker β‐galactosidase in both WCF and PCF: as shown in Figure 1B, both cell types showed a linear increase in the staining intensity for β‐galactosidase at increased passages in culture (Figure 1C), a difference that became significant between P1 and P9 (*P* < .001 and <.05 in WCF and PCF, respectively). Moreover, this trend was dependent from the genotype: indeed, PCF showed a significant (*P* < .05) lower percentage of β‐galactosidase compared to WCF.

### 3.2 DNA damage in *Apc* mutated colon fibroblasts

**FIGURE 2:**
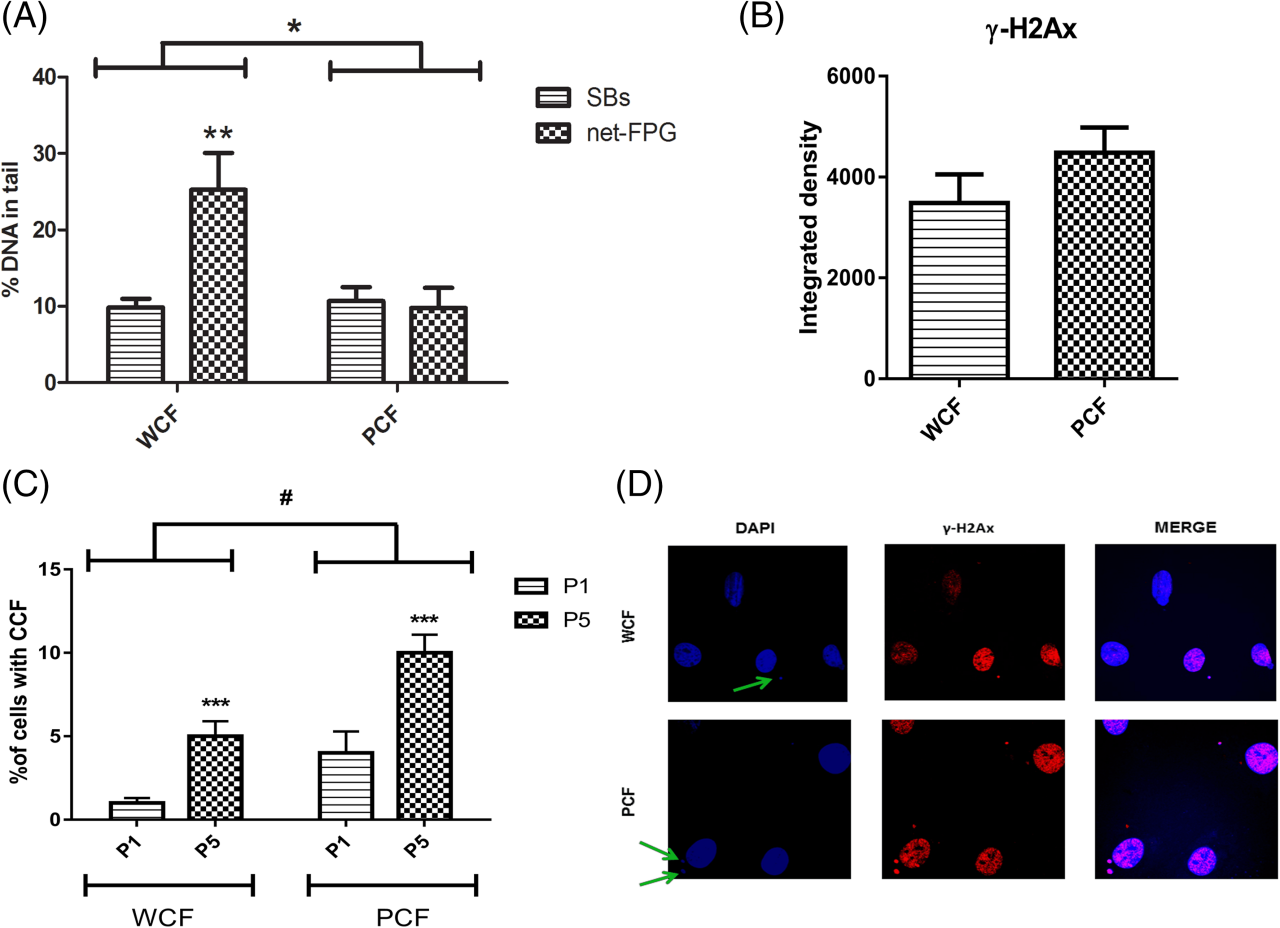
DNA damage and Cytoplasmic chromatin. (A) DNA strand breaks (SBs) and FPG sites (oxidized bases, Net FPG) measured by comet assay on three couples of matched isolated WCF and PCF between P2‐6 (mean ± SE, n = 2 independent experiments). * *P* < .05 significantly different from WCF and ** *P* < .01 significantly different from SBs. (B) Integrated density calculated on IF images of γ‐H2Ax performed on three couples of matched WCF and PCF (mean ± SE, n = 10 microscopic field for both groups). (C) % of cells bearing cytoplasmic chromatin foci determined in 10 microscopic fields/slide (40X magnification) in PCF and WCF between passage 1 and 5 (mean ± SE, n = 17 and 15 slides for WCF and PCF, respectively). # *P* < .05 significantly different from WCF; *** *P* < .001, significantly different from P1. (D) Representative images of γ‐H2Ax captured with confocal microscope (×100 magnification). Green arrows indicate cytoplasmic chromatin foci [Color figure can be viewed at wileyonlinelibrary.com]

The standard version of the comet assay was performed to detect DNA strand breaks (SBs) in primary fibroblast cultures. Oxidized DNA bases were also assessed with the modification of the method involving the use of the enzyme FPG. Two‐way ANOVA analysis highlighted that the amount of DNA damage, SBs and net FPG sites, was significantly dependent on genotype (*P* < .05): indeed, PCF exhibited almost the same levels of SBs and oxidative damage (net FPG) whereas oxidative damage in WCF was higher than in PCF (*P* < .05, Figure 2A). In agreement with the result on SBs, the analysis of the phosphorylated histone 2Ax (γ‐H2Ax), a marker of DNA breakage performed on IF images did not show differences between PCF and WCF (Figure 2B). As cytoplasmic chromatin foci have been previously detected in both senescent and cancer cells, deriving from DNA damage in the former, and chromosome segregation errors or DNA fork stall or collapse after replication stress in the latter,^26^ we decided to evaluate the presence of cytoplasmic chromatin foci in γ‐H2Ax‐stained slides (Figure 2D). Considering P1 and P5 separately, two‐way ANOVA analysis outlined that the presence of cytoplasmic chromatin foci was dependent on the genotype (*P* < .05) but also that passages in culture affected the percentage of cytoplasmic chromatin foci, which was significantly increased (*P* < .001) at P5 compared to P1 in both PCFs and WCFs (Figure 2C).

### 3.3 Inflammatory phenotype in *Apc* mutated colon fibroblasts

**FIGURE 3:**
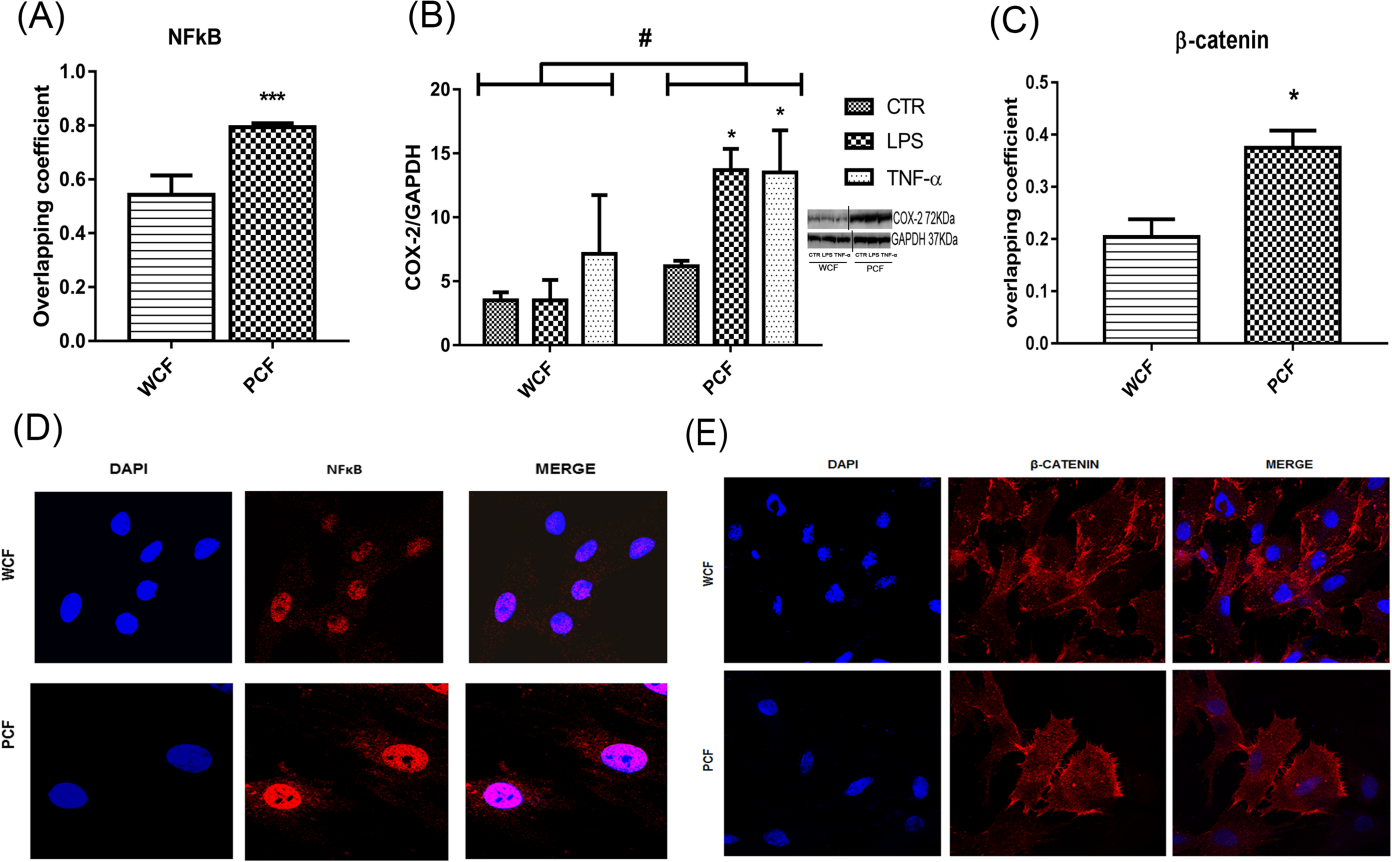
Inflammatory phenotype in PCF and WCF. (A) IF assay was performed on WCF and PCF between P1‐6 to assess the nuclear translocation of NFkB which was expressed as overlapping coefficient between NFkB signal and nuclear DAPI on three couples of age matched isolated WCF and PCF fixed at different passages (mean ± SE, n = 12 for WCF and n = 15 for PCF) *** *P* < .001. (B) COX‐2 expression in WCF and PCF and representative blots from different parts of the same membrane (mean ± SE, n = 3 independent experiments). * *P* < .05 significantly different from control (CTR); ^#^ *P* < .05 significantly different from WCF. (C) IF to assess nuclear translocation of β‐catenin in PCF and WCF at P5‐6 was performed and results expressed as for NFkB (mean ± SE, n = 3 for both cell lines) * *P* < .05. Panels (D) and (E): representative IF images for NFkB and β‐catenin, respectively (×100 magnification) [Color figure can be viewed at wileyonlinelibrary.com]

Considering that an inflamed microenvironment promotes carcinogenesis, we assessed whether PCF cells exhibited activation of inflammatory pathways, the most studied of which is the NFkB signaling pathway. To this aim, NFkB IF was performed on PCF and WCF at different passages and images analysis carried out as described above. Based on the values of the overlapping coefficient (comprised between 0 and 1, meaning 0% and 100% overlapping of NFkB signal, red, and nuclear DAPI, blue), we found that PCF had higher levels of NFkB nuclear translocation compared to WCF (*P* < .001, Figure 3A,D) indicating that this pathway is overactivated in mutated cells. To confirm this hypothesis, we measured the levels of COX‐2, whose expression is under control of NFkB^27^: WCF and PCF were treated for 24 h with LPS or TNF‐α as described above, then COX‐2 protein expression was measured by western blotting. As shown in Figure 3B, PCF showed a significantly higher expression of COX‐2 compared to WCF in basal conditions together with an enhanced response to inflammatory stimuli as outlined by two‐way ANOVA (*P* < .05 in both cases), confirming the upstream hyperactivation of the NFkB pathway. Finally, in order to further confirm that constitutive NFkB activation was strictly linked to *Apc* mutation in PCF, we verified the activation of Wnt signaling by nuclear translocation of β‐catenin: indeed, NFkB is a downstream target of Wnt.^28^ Similarly to NFkB, β‐catenin was significantly more translocated in PCF nuclei compared to WCF (*P* < .05, Figure 3C,E) in basal condition, so confirming the hyperactivation of Wnt signaling in *Apc* mutated fibroblasts.

### 3.4 Ex‐vivo exposure of Pirc tumor samples to colon fibroblast‐conditioned media

An ex‐vivo system consisting in maintaining in culture for 24 h adenoma samples from Pirc old rats was previously used in our group.^22^ Consequently, we thought it of interest to use the same system to assess the effects of PCF and WCF conditioned media on adenoma inflammatory state, evaluating COX‐2 protein expression. COX‐2 was increased in adenomas cultured with WCF‐conditioned media and a slight further increase was observed for the ones incubated with PCF media: however, these differences were not statistically significant (Figure S1). Consequently, this result does not rule out a possible role of colon fibroblasts in releasing factors supporting inflammation.

### 3.5 CAFs markers evaluation in *Apc* mutated colon fibroblasts

**FIGURE 4:**
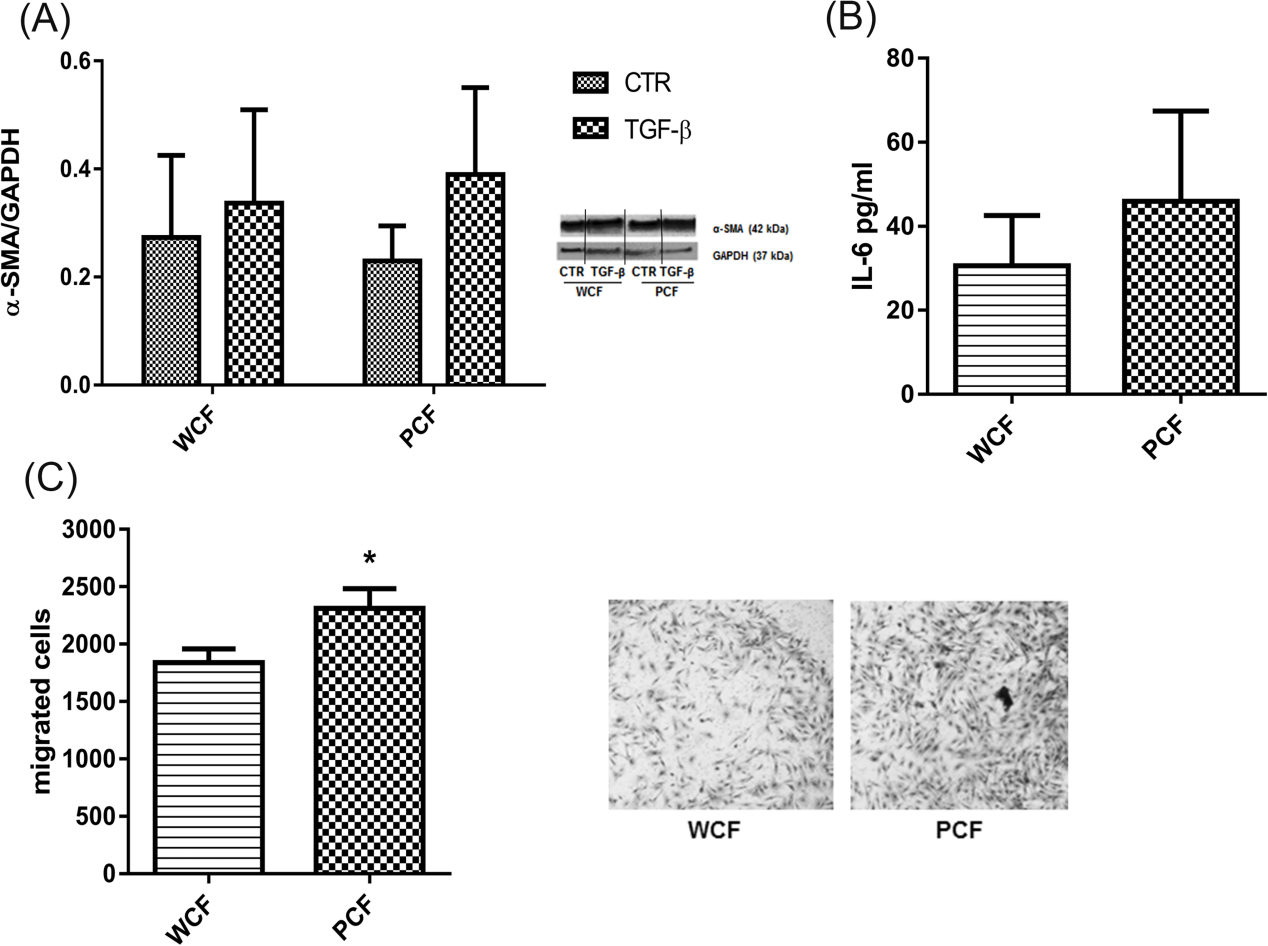
Assessment of CAFs markers in PCF and WCF. α‐SMA (A) and IL‐6 (B) were measured by western blotting and ELISA assay in whole cells protein extracts and cell‐conditioned media respectively (mean ± SE, n = 3 independent experiments). Representative blots from different parts of the same membrane are shown (A). (C) Invasion test performed by means of the Boyden chamber assay. Cells (15 000) were seeded on Matrigel coated filters and fixed 24 h later. Stained filters (representative images on the right side of the panel) were mounted on microscope slides and cells counted on the whole surface (×40 magnification). Bars are means ± SE, n = 3 independent experiments, * *P* < .05 [Color figure can be viewed at wileyonlinelibrary.com]

Since PCFs demonstrated to be endowed with a proinflammatory phenotype, we decided to investigate the possibility that they already showed features of activated fibroblasts. The expression of α‐SMA was assessed in both WCF and PCF in basal conditions and after a 24 h treatment with TGF‐β (10 ng/mL), a well‐known fibroblast‐activating factor. Moreover, we measured the release of IL‐6 in both fibroblast culture media. As shown in Figure 4A,B, we did not find significant differences between WCF and PCF for either parameter. More in detail, considering the expression of α‐SMA, there was a slight and not significant response to TGF‐β stimulation. In addition, we did not find differences in IL‐6 levels measured in culture media from WCF and PCF cells in basal conditions.

On the other hand, the invasion assay in Boyden chamber showed that PCF featured significantly (*P* < .05) higher spontaneous invasiveness (ie, in absence of chemoattractant or FBS gradient) compared to WCF (Figure 4C). Consequently, these results suggest that PCF show some features of activated fibroblasts although they do not overexpress CAFs peculiar markers such as IL‐6 and α‐SMA.

### 3.6 Microarray on normal mucosa from wt and Pirc rats at 1 month of age

**FIGURE 5:**
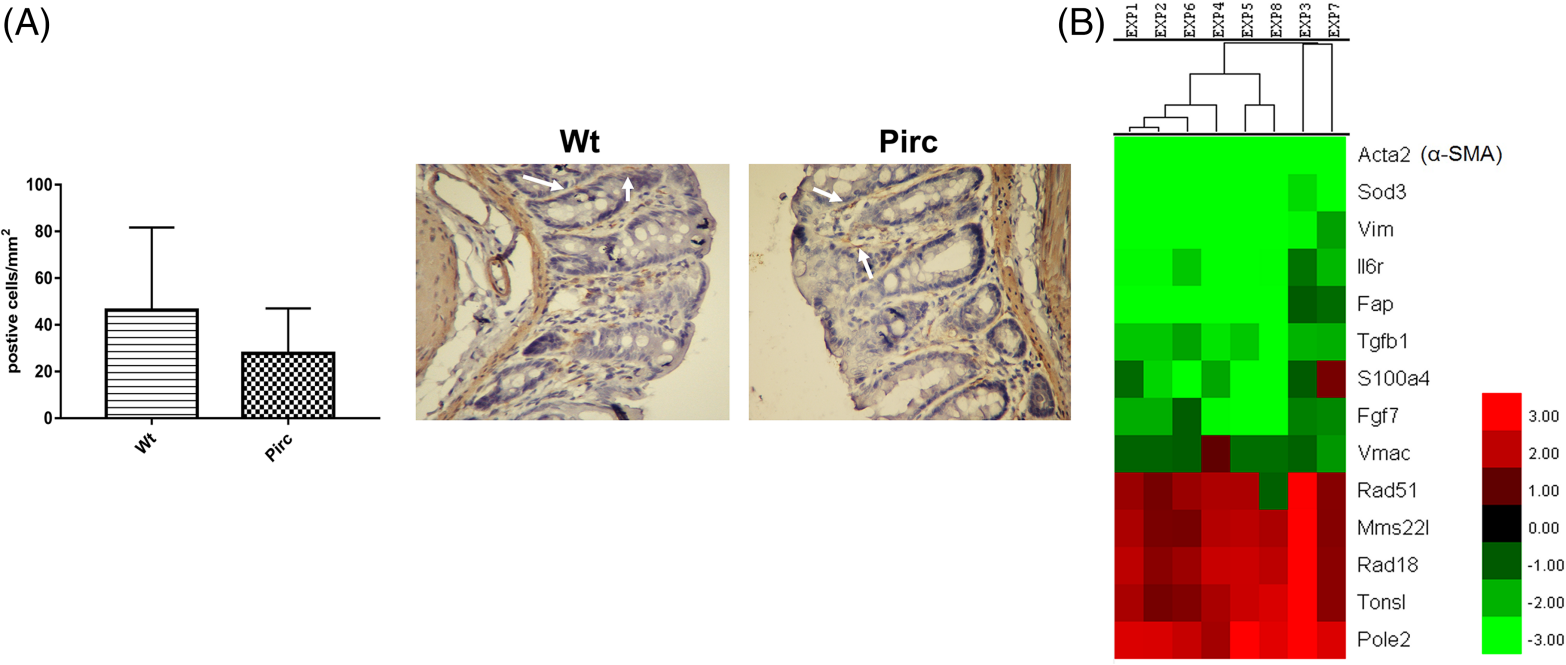
α‐SMA‐positive cells and expression profiles of genes of interest in colon mucosa of one‐month old rats. (A) IH for α‐SMA was performed on normal mucosa sections from Pirc and wt rats (n = 3 for both). Positive cells were counted from two independent and blinded operators and results were expressed as positive cells/mm^2.^(mean ± SE). Representative images are shown on the right side of the graph (×40 magnification). (B) Hierarchical cluster analysis performed on genes of interest, found to be differentially expressed in the normal mucosa of 8 Pirc rats compared to a single pool of RNA from the normal mucosa of wt rats. Red indicates upregulation while green indicates down‐regulation in Pirc vs wt [Color figure can be viewed at wileyonlinelibrary.com]

Considering the results obtained on CAFs markers (IL‐6 and α‐SMA), we decided to analyze the differences in gene expression of markers of activated fibroblasts and the related stimuli needed for their activation. This analysis was performed by whole genome array on normal mucosa samples from 1‐month‐old Pirc and wt rats. Interestingly (Table 1 and Figure 5B), *Vimentin* and *Acta2* (α‐Sma gene) were strongly downregulated in Pirc normal mucosa compared to wt (*P* < .01 and <.001, respectively), as was Fibroblast activating protein (*Fap*) and *S100a4* although, in these case, the difference was only nearly significant (*P* = .077 and .061, respectively). In line with gene expression, we found both less α‐SMA‐positive cells in Pirc normal mucosa sections compared to wt (positive cells/mm^2^, Figure 5A) and reduced protein expression evaluated by western blotting (data not shown), although the differences were not statistically significant in either case. Moreover, we observed lower expression of *Tgf‐β* (*P* < .001), and *Fgf7* (*P* = .07), a well‐known fibroblast activating signal, in Pirc normal mucosa compared to wt. Consequently, these data suggest the absence, at 1 month of age, of adequate stimuli from epithelial cells to activate surrounding fibroblasts, and support the *in vitro* observations on the very low protein expression of markers of activated fibroblasts, α‐SMA and IL‐6 (Figure 4A,B, respectively). Indeed, IL‐6 receptor (*Il6r*) also resulted to be significantly down‐expressed in Pirc normal mucosa (*P* < .01).

**TABLE 1:** Genes of interest, listed with the respective mean fold change (positive or negative values indicating upregulation or downregulation in Pirc vs wt)

ProbeName	GeneName	SystematicName	Protein name	Mean	SE	*P*
A_64_P140716	Acta2	NM_031004	α‐SMA	−8.23	0.78	.00026
A_44_P470430	Fap	NM_138850	Fibroblast activating protein	−3.87	1.05	.07724
A_42_P509365	Vim	NM_031140	Vimentin	−3.86	0.75	.01220
A_42_P772157	Il6r	NM_017020	IL‐6 receptor	−2.54	0.56	.01587
A_42_P522899	Tgfb1	NM_021578	TGF‐β1	−2.13	0.29	.00635
A_44_P392234	Fgf7	NM_022182	Fibroblast growth factor 7	−1.73	1.14	.07826
A_64_P032405	Vmac	NM_001001720	Vimentin associated coiled‐coil protein	−1.11	0.29	.02869
A_43_P11489	S100a4	NM_012618	S100 calcium‐binding protein A4 (also known as Fibroblast Specific Protein 1, FSP‐1)	−2.13	0.63	.06130
A_44_P231935	Rad51	NM_001109204	RAD51	1.51	0.58	.13981
A_64_P156947	Mms22l	NM_001135780	MMS22‐like, DNA repair protein	1.60	0.48	.03798
A_64_P104988	Tonsl	NM_001130572	Tonsoku‐like, DNA repair protein	1.61	0.45	.04239
A_43_P23316	Rad18	NM_001077673	RAD18	1.81	0.48	.02488
A_44_P236738	Pole2	NM_001169108	Polimerase epsilon 2	2.30	0.61	.01864
A_43_P11601	Sod3	NM_012880	Superoxide dismutase 3	−6.00	0.80	.00119

Additionally, the lower oxidative DNA damage found in PCF compared to WCF, would suggest a possible hyperactivation of DNA damage repair systems (DDR), particularly BER, involved in oxidative damage repair. Interestingly, DNA polymerases ε subunit 2 (*Pole2*), involved in the repair of 8‐oxoG^29^ was significantly overexpressed (*P* < .01) in Pirc normal mucosa compared to wt at age 1 month. Furthermore, the presence of cytoplasmic chromatin foci that we found could be at least in part attributed to the resolution of replication stress by overcoming DNA fork stall. So, we decided to look at the expression of genes involved in the DNA Damage Tolerance system. MMS22L/TONSL is the sensor of the DNA Damage Tolerance response which recognizes stalled forks: interestingly, both *Mms22l* and *Tonsl* genes were overexpressed in Pirc normal mucosa compared to wt at 1 month of age (*P* < .05 for both genes). This complex recruits in turn Rad18 and Rad51 that allow the repair of DNA breaks occurred because of DNA fork stall^30^: of note, the former was overexpressed in Pirc normal mucosa compared to wt (*P* < .05) while the latter followed the same trend but the difference was not statistically significant.

## 4 DISCUSSION

Tissue microenvironment modifications occur during CRC and are mainly driven by the activation of normal tissue resident fibroblasts into CAFs accounting for a worse prognosis.^11^ The role of fibroblast *APC* mutation in this context is not clear, and few data on the characterization of colon fibroblasts mutated in *APC*, mostly isolated from FAP patients, are available. These considerations led us to study the phenotype of *Apc* mutated colon fibroblasts derived from 1 month aged Pirc rats (*Apc* ^*+/−*^ *)*: at this age, there are no macroscopic lesions or MDFs detectable in the colon of this genetic CRC model, but molecular changes promoting CRC are already ongoing.^18^ Furthermore, the use of this genetic model represents the only possibility to study *Apc*‐driven cellular mechanisms in very early stages of CRC: this is quite impossible using human colon biopsies both from patients (already bearing polyps) and healthy individuals.

### 4.1 Proliferation and replicative senescence in *Apc* mutated colon fibroblasts

As altered Wnt pathway accounts for hyperproliferation in *Apc* mutated cells,^1^ we expected to find higher proliferative activity in PCF compared to WCF: indeed, PCF showed a nearly double proliferation rate compared to WCF at initial passages in culture (1 ≤ *P* ≤ 6). With increasing passages in culture, proliferative rates slowed down in both cell types; concomitantly, we observed a linear increase in positivity to senescence associated β‐galactosidase marker, more pronounced in WCF than in PCF. Furthermore, β‐galactosidase‐positive cells could be found in WCF at intermediate passages in culture (P4‐5) but were rare in PCF at this time point. Thus, replicative senescence was approached in both PCF and WCF at 7 ≤ *P* ≤ 9, in line with the limited replicative potential of normal cultured fibroblasts from rats, ranging around 15 passages before full senescence onset.^31^ However, *Apc* mutation slowed down replicative senescence, thus conferring to cells an advantage to live longer.

### 4.2 DNA damage in *Apc* mutated colon fibroblasts

Cells can also enter senescence in response to elevated and unresolved DNA damage^32^: in accordance with this scenario, we tried to verify whether the senescence onset observed in WCF and, more slowly, in PCF could be at least partly due to elevated DNA damage. Performing the comet assay on PCF and WCF between P4‐8, we found comparable amounts of SBs, and these data were confirmed quantifying by IF the levels of γ‐H2Ax. Instead, a significantly higher oxidative DNA damage level was observed in WCF compared to PCF by the use of FPG (which recognizes 8‐oxoG sites like OGG1 repair enzyme^33^) in the modified comet assay. This difference could be attributed to oxidative stress resistance in PCF, similar to what happens in tumor cells. Indeed, several authors have reported that tumor cells initially maintain low levels of intracellular ROS through antioxidant systems activation,^34^, ^35^ increased oncogenic activity and/or decreased tumor suppressor activity.^36^, ^37^ Besides, previous analyses performed by our group^18^ on normal mucosa of Pirc and wt rats of 1 month of age reported higher expression of *Apex1* and *Ogg1* in Pirc rats: the latter is a component of BER system and repairs 8‐oxoG adducts.^29^ Consequently, the lower levels of oxidative damage found in mutated PCF compared to WCF could be at least in part attributable to Ogg1 activity and more generally to the lack of BER system inhibition from Apc. Notably, mutated *Apc* lacks the DNA Repair Inhibitory domain (DRI), which blocks the BER system.^38^ This hypothesis is further sustained by the significantly higher expression of DNA Pol ε, a component of BER, found in the present work in Pirc normal mucosa at 1 month of age (Table 1). Thus, it is conceivable that increased levels of DNA repair enzymes together with the lack of inhibition of BER system in PCF account for the reduced DNA oxidative damage observed in PCF compared to WCF. Moreover, unresolved oxidative DNA damage in WCF could contribute to their early entry into senescence compared to PCF, so further enforcing the possible advantage conferred by mutated *Apc* on cell survival and lifespan.

### 4.3 Cytoplasmic chromatin foci in *Apc* mutated colon fibroblasts

Colon fibroblasts tended to develop cytoplasmic chromatin foci while progressing towards senescence onset, in line with data reported in literature.^26^ However, it was intriguing to note that their percentage at all passages was higher in PCF, despite their delay in entering senescence (see above results on β‐galactosidase).

Apart from their association with senescence, cytoplasmic chromatin foci can be also originated during mitosis, particularly during interphase, from damaged DNA regions, or can be the consequence of DNA replication forks stall or collapse resolution as observed in cancer cell lines.^39^, ^40^, ^41^ Alterations in replication rate can lead to replication stress that is characterized by DNA forks stall or collapse.^42^ The MMS22L/TONSL complex mediates the recovery from stall recruiting proteins involved in homologous repair (HR) system^43^ and it has also been implicated in carcinogenesis.^44^, ^45^ As PCF show increased replication rates compared to WCF, it is possible that they are in a state of replicative stress. In line with this hypothesis, array data obtained on colon normal mucosa of 1‐month‐old Pirc and wt rats showed overexpression of the DNA Damage Tolerance and HR components *Mms22l* and *Tonsl*, *Rad18* and *Rad51*, respectively, in Pirc normal mucosa compared to wt. It is thus possible that cytoplasmic chromatin foci in PCF derive from DNA repair activity mechanisms involving the DNA Damage Tolerance response.

### 4.4 Inflammatory and CAFs phenotype in *Apc* mutated colon fibroblasts

Nuclear translocation of NFkB is associated with the activation of several processes which in turn promote carcinogenesis.^46^ Furthermore, the role of cytoplasmic chromatin foci in mediating inflammation through c‐GAS‐STING pathway and consequently NFkB activation has been demonstrated.^26^ We found a significantly higher nuclear translocation of NFkB in PCF compared to WCF, associated with elevated presence of cytoplasmic chromatin foci as outlined above. Concomitantly, we found a significantly higher expression of the proinflammatory marker COX‐2 in PCF compared to WCF, accounting for the higher sensitivity exhibited by PCF to proinflammatory stimuli as showed by COX‐2 expression increase in response to LPS and TNF‐α stimulation: the different response to those stimuli was significantly dependent from the genotype. Moreover, nuclear translocation of β‐catenin was also significantly higher in PCF, as expected in an *Apc* mutated context, confirming that NFkB hyperactivation in PCF is a consequence of Wnt signaling.^28^ These results suggest that *Apc* mutation contributes to sustain an early inflammatory phenotype in colon fibroblasts which in turn could be further maintained by cytoplasmic chromatin foci via NFkB continuous activation.

Nonetheless, neither IL‐6 release was drastically changed in PCF‐conditioned media, nor COX‐2 levels were significantly modified in tumor samples cultured for 24 h with the same media, thus suggesting the lack of adequate signals from PCF, at this stage, to induce and sustain inflammation in colon tissue microenvironment, despite their inflammatory features.

PCF demonstrated significantly higher spontaneous invasiveness compared to WCF, a characteristic proper of CAFs. However, α‐SMA expression was not increased in PCF compared to WCF even after stimulation with TGF‐β, and microarray on 1‐month‐old normal mucosa showed a significantly lower expression of *Acta2* (α‐SMA gene*)* in Pirc versus wt, further confirmed by a lower amount of α‐SMA‐positive cells found in Pirc normal mucosa by means of immunohistochemistry. The expression of additional markers of activated fibroblasts, such as *Vimentin* and *Fap*, and the activating molecules *Tgf‐β* and *FgF7* ^47^ was also reduced in Pirc colon mucosa. Nonetheless, the high expression of the specific fibroblast marker *S100a4* ^47^ suggests that fibroblasts are quantitatively well represented in our specimens.

These observations led us to hypothesize that *Apc*‐mutated colon fibroblasts isolated from 1‐month‐old Pirc rats already show a constitutive inflammatory phenotype but need further signals to acquire a fully activated phenotype which in turn could allow them to produce an inflammatory environment in colon tissue. This hypothesis is in line with some, although still limited, evidences about the tumor suppressing activity of a subpopulation of CAFs presenting low‐levels of α‐SMA^48^ whose behavior switches into protumorigenic in response to specific stimuli. This could be the case of PCF which could limit CRC until specific stimuli are released from the surrounding epithelial cells.^49^, ^50^

## 5 CONCLUSIONS

All considered, the present study highlights that *Apc* mutated colon fibroblasts isolated from normal mucosa in absence of apparent precancerous lesions, show inflammatory features *per se*, in absence of any specific stimulus as demonstrated by the constitutively higher expression of COX‐2 protein. Moreover, *Apc* mutated fibroblasts show a significantly higher sensitivity to inflammatory stimuli (LPS, TNF‐α) compared to wt counterparts. On the other hand, they seem to be nonfully activated fibroblasts: indeed, they are endowed with higher proliferative rates, late replicative senescence onset and higher invasiveness but they do not express CAF markers. Despite this partial activation, lower levels of oxidative DNA damage suggest that *Apc* mutation could confer to these colon fibroblasts resistance to oxidative stress as previously suggested by our group and other authors.^15^, ^16^, ^18^ Additionally, the presence of cytoplasmic chromatin foci, showed here for the first time in *Apc* mutated cells, suggests resistance to replicative stress, and can be responsible for constitutive activation of NFkB signaling in Pirc fibroblasts. Usually, these features confer to malignant cells selective advantages: consequently, it is conceivable that they could make mutated colon fibroblasts supportive of both CRC and desmoid tumors since very early stages in FAP patients, thus suggesting the possibility to investigate the use of antiinflammatory drugs in young diagnosed FAP patients without any polyps detected yet. Indeed, 20% of FAP patients die for complications due to intraabdominal desmoid tumors also in case of a previous colectomy.^4^

On the whole, these data show for the first time that a proliferative and inflammatory phenotype characterizes *Apc* mutated colon fibroblasts at very early stages of colon carcinogenesis, favoring the establishment of a protumorigenic environment for preneoplastic lesion development. Future studies will have to clarify the influence of the cross‐talk with epithelial cells to acquire additional features supporting CRC, possibly using more complex *Apc* deficient *in vitro* systems. Additionally, it will be important to deepen the knowledge on the role of *Apc* protein in DNA repair and oxidative stress resistance, and its interplay with the immune‐surveillance pathway activated by cytoplasmic chromatin foci (namely cGAS‐STING): all these are key processes for cancer cell survival, and can provide new possible targets for therapy or prevention.

## CONFLICT OF INTERESTS

The authors declare no conflicts of interest.

## ETHICS STATEMENT

All procedures involving animals were performed in accordance to EU Directive 2010/63/EU for animal experiments and ARRIVE guidelines following an approved protocol.

## Supporting information


**Appendix S1** Supporting Information

## Data Availability

The microarray data sets supporting the results of this article are available in the MIAME public database ArrayExpress repository [http://www.ebi.ac.uk/arrayexpress/] (Accession Number E‐MTAB‐10016). Further details and other data that support the findings of our study are available from the corresponding author and/or first author upon request.
