# Supplementary material for: Colon fibroblasts from Pirc rats (F344/NTac‐Apc am1137) exhibit a proliferative and inflammatory phenotype that could support early stages of colon carcinogenesis
Source: Int J Cancer. 2021 Sep 18;150(2):362–73. doi: 10.1002/ijc.33796 (PMC9291568; doi:10.1002/ijc.33796)
Supplement: Supplementary file 1 — Appendix S1 Supporting Information [file IJC-150-362-s001.pdf]

Supplementary Materials for the Manuscript

Colon fibroblasts from Pirc Rats (F344/NTac-*Apc* ^{am1137}) exhibit a proliferative and inflammatory phenotype that could support early stages of colon carcinogenesis

Katia Tortora, Francesca Margheri, Cristina Luceri, Alessandra Mocali, Sara Ristori, Lucia Magnelli, Giovanna Caderni, Lisa Giovannelli

Supplementary Material & Methods........ 2

Supplementary figure 1........ 4

Supplementary References 5

Supplementary Material & Methods

Immunocytofluorescence (IF) and immunohistochemistry (IH)

For IF assay cells were seeded (10000 cells/well) onto round glass coverslips in 24-MW plates. The assay and following capture of images using a Bio-Rad MRC 1024 ES Confocal Laser Scanning Microscope (Bio-Rad, Hercules, CA) equipped with a 15 mW Krypton/Argon laser source for fluorescence measurements were performed as described elsewhere.[1] Antibodies used were: anti phospho-histone H2Ax (#2577, Cell Signaling) 1:100; NFkB(p65) (sc-109 rabbit, Santa Cruz Biotech, INC) 1:100; β-catenin (610154 mouse, BD bioscience) 1:400; anti-rabbit IgG (Alexa Fluo 594) 1:500. Ten microscopic fields/slide (γ-H2Ax ones) were counted for the presence of cytoplasmic chromatin foci under a fluorescent microscope (40X magnification) and the result expressed as the % of cells with cytoplasmic chromatin foci. Immunohistochemistry (IH) for α-SMA on normal mucosa sections (5 µm thickness) from one month old rats, was performed as previously described.[2]

Ex-vivo of Pirc adenomas in conditioned medium from PCF and WCF

Ex-vivo system of adenomas samples from 11 months old Pirc rats (n=2) were established as previously described.[2] For the aim of this study, adenoma samples were maintained in culture in 48-MW plates for 24 h with conditioned media from PCF or WCF or with unconditioned one. At the end of the incubation, samples were washed with 1X PBS and proteins were extracted as described below. Conditioned media from both cultures were collected from confluent flasks, centrifuged at 1200 rcf for 5', aliquoted in 1.5 ml tubes and stored at -20°C until use.

Western blotting assay on PCF and WCF after pro-inflammatory stimulation

PCF and WCF were seeded in 6-MW plate (50000 cells/well) and treated for 24 h with advanced DMEM supplemented with 1% FBS, 1% L-Glutamine, 1% antibiotic-antimycotic and: a) 10 µg/ml LPS; b) 20 ng/ml TNF-α; or c) 10 ng/ml TGF-β. Treatments started when cells were at 70% confluence; at the end of the treatments, proteins from monolayers, normal mucosa and *ex vivo* samples were extracted, run and bands acquired and quantified as previously described.[2,3] Each measured density was normalized by using the corresponding GAPDH density value. The antibodies used were: COX-2 (160126 rabbit, Cayman Chemical), 1:200; PCNA (PC10: sc-56 mouse Santa Cruz Biotechnology, INC), 1:1000; NFkB(p65) (sc-109 rabbit, Santa Cruz Biotech, INC); GAPDH (14C10 Rabbit mAb Cell Signaling); α-SMA antibody (ab5694, Abcam) 1:500; 1:3000 anti-rabbit IgG HRP-linked antibody (#7074 Cell Signaling), 1:4000; anti-mouse IgG- peroxidase (A9044 Sigma-Aldrich, Milan, Italy), 1:5000.

Comet assay

The comet assay was performed in order to assess basal levels of DNA breaks and DNA base oxidation in PCF and WCF. Cultured cells were detached by trypsin incubation for not more than 5’ and counted in a Burker chamber. Then, 20 x 10^4 cells were embedded in 70 µl of 0.75% low melting point agarose (LMA) and layered on pre-coated (1% normal melting agarose, NMA) microscope slides (2 gels/slide), covered with 22x22 mm coverslips and let solidify for 10’ at 4°C; for each sample, 3 slides were prepared. The standard and FPG-modified version of the comet assay for the detection of oxidized bases, and the following scoring and data elaboration were performed as previously described.[4]

Supplementary figure 1

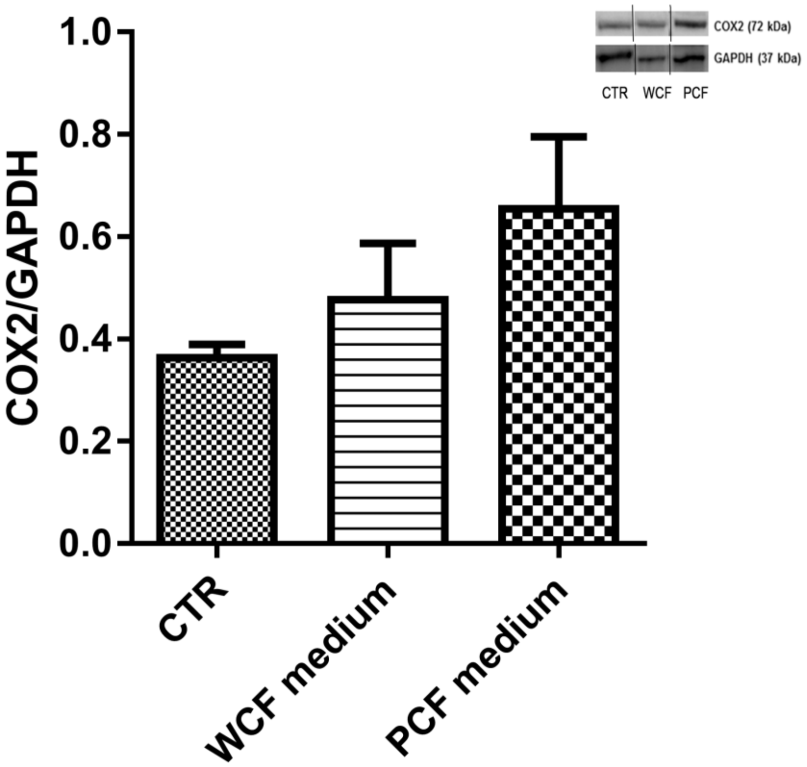

Supplementary figure 1. *Apc* mutated tumors from Pirc rats cultured *ex vivo* with fibroblast-conditioned media. Tumor samples from 11 month Pirc male rats were maintained in culture for 24h with standard medium or with conditioned media from WCF or PCF, then COX-2 level was assessed by western blotting (mean ± SE, n=3 independent experiments). Representative blots from different parts of the same membrane are presented.

Supplementary References

1. Laurenzana A, Margheri F, Biagioni A, Chillà A, Pimpinelli N, Ruzzolini J, Peppicelli S, Andreucci E, Calorini L, Serratì S, Del Rosso M, Fibbi G. EGFR/uPAR interaction as druggable target to overcome vemurafenib acquired resistance in melanoma cells. *EBioMedicine* 2019;
2. Tortora K, Femia AP, Romagnoli A, Sineo I, Khatib M, Mulinacci N, Giovannelli L, Caderni G. Pomegranate By-Products in Colorectal Cancer Chemoprevention: Effects in Apc-Mutated Pirc Rats and Mechanistic Studies In Vitro and Ex Vivo. *Mol Nutr Food Res* 2018;62.
3. Pitozzi V, Mocali A, Laurenzana A, Giannoni E, Cifola I, Battaglia C, Chiarugi P, Dolara P, Giovannelli L. Chronic resveratrol treatment ameliorates cell adhesion and mitigates the inflammatory phenotype in senescent human fibroblasts. *Journals Gerontol - Ser A Biol Sci Med Sci* 2013;
4. Ladeira C, Koppen G, Scavone F, Giovannelli L. The comet assay for human biomonitoring: Effect of cryopreservation on DNA damage in different blood cell preparations. *Mutat Res - Genet Toxicol Environ Mutagen* 2019.
